# Composite grafts for fingertip amputations: A systematic review protocol

**DOI:** 10.1016/j.isjp.2019.05.001

**Published:** 2019-05-23

**Authors:** Mimi R. Borrelli, Madeleine L. Landin, Riaz Agha, Aina Greig

**Affiliations:** a Hagey Laboratory for Pediatric Regenerative Medicine, Department of Surgery, Division of Plastic Surgery, Stanford University School of Medicine, Stanford, CA, United States; b King’s College London, Guy’s, King’s and St Thomas’ School of Medicine, Guy’s Campus, Great Maze Pond, London SE1 9RT, United Kingdom; c Plastic Surgery Department, Chelsea and Westminster Hospital NHS Foundation Trust, 369 Fulham Rd, Chelsea, London SW10 9NH, United Kingdom; d Plastic Surgery Department, St Thomas’ Hospital, Guy’s and St Thomas’ Hospital NHS Foundation Trust, Westminster Bridge Road, London SE1 7EH, United Kingdom

**Keywords:** Composite graft, Distal finger tip, Amputation, Reconstruction

## Abstract

**Background:**

There is much debate in the hand surgery literature as to the management of fingertip amputations. Much research continues to be published in this area. Methods of reattachment include microsurgical and non-microsurgical (composite graft) replantation. The role of composite grafts lacks clarity in terms of outcomes, success rates and complications. Hence there is a need for an evidence synthesis, which can guide patient selection, the consent process and determine graft survival rates and functional outcomes to optimise patient outcomes.

**Methods:**

Search of the databases OVID MEDLINE, PubMed, EMBASE, SCOPUS, The Cochrane Library and clinical trial registries from inception using terms “fingertip” “digital tip” “digit” “finger” “thumb” “amputation” “replantation” “reattachment” “reimplantation” and “composite graft” as key terms with “AND” selected as a Boolean operator, limited to humans will be conducted by two independent researchers. The patient population will include adults and children. Studies will be included if they report: (1) primary data; (2) outcomes of ‘composite grafts’ or ‘nonmicrosurgical replantations’; (3) graft survival, (4) 5 or more cases. Articles will be excluded if surgical techniques involve: (1) composite graft pocketing, or (2) microsurgical replantation or (3) additional flaps (pulp or local). Full exclusion and inclusion criteria are described within this protocol. Data extraction will include; demographic details, patient comorbidities, amputation nature and level, functional, and aesthetic outcomes, complications and need for secondary procedures. All data extracted will be cross-checked, and discrepancies resolved through consensus.

**Dissemination:**

This review will be published in a peer-reviewed journal and will be presented at national and international conferences to inform the practice of other clinicians.

## Highlights

• There is a lack of evidence for composite grafting for distal finger tip amputation.
• We present a systematic review protocol on the use of composite grafts.
• This review will aim to provide clinicians with more guidance.

## 1 Introduction

**Fig. 1:**
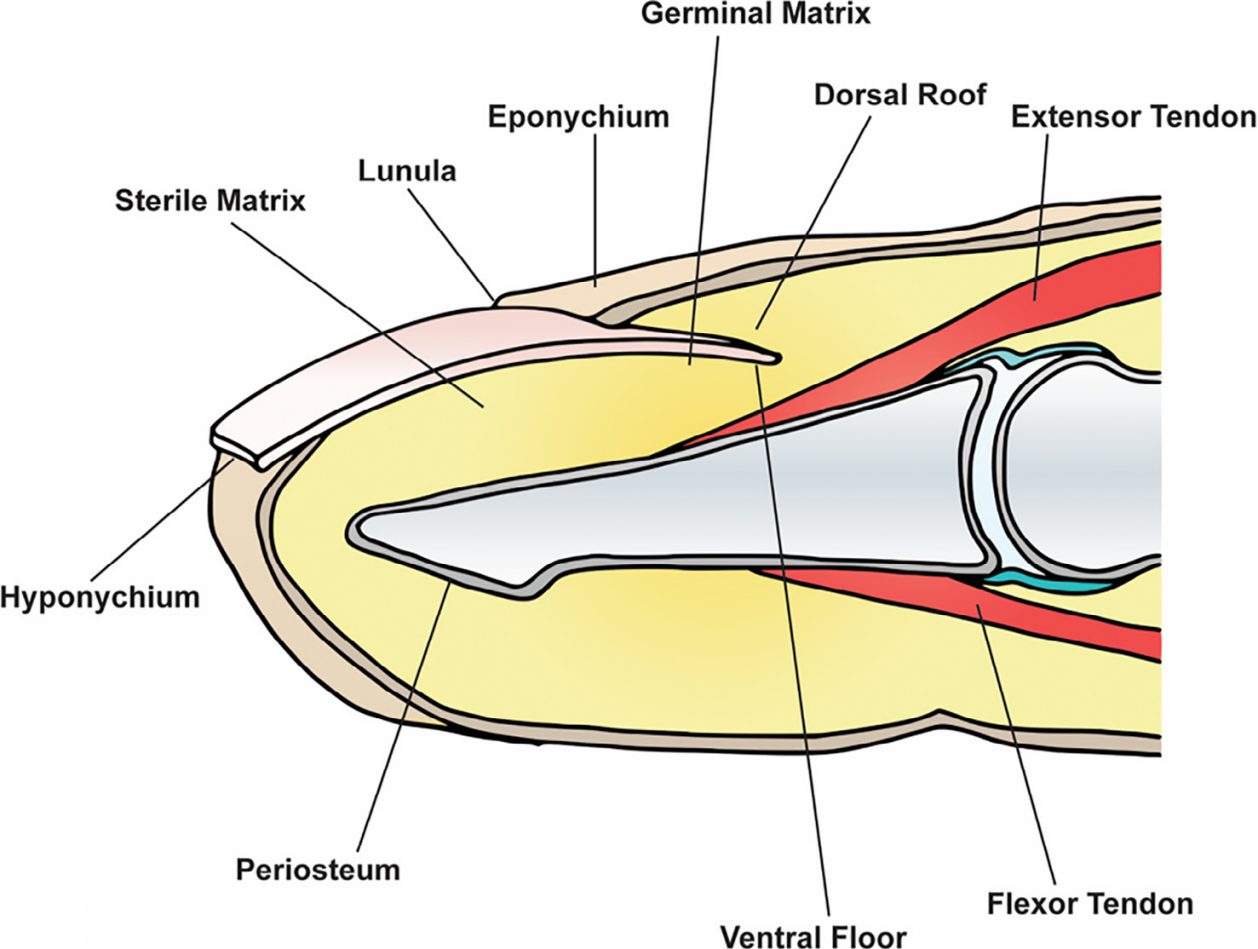
Lateral view of the distal finger showing the key anatomical fingertip landmarks.

The fingertip is the segment distal to the insertion of the flexor and extensor tendons on the distal phalanx [1] (Fig. 1). A fingertip amputation is the loss of a part of a finger distal to the level of the distal interphalangeal joint (DIPJ). It is a common presentation to the emergency department. In the paediatric population it often occurs following crush injuries from doors [2], [3]. Fingertips amputations can cause pain, disturbances to sensation, fine motor dexterity, nail growth, and the aesthetics of the hand, which may result in significant psychological distress [4]. Treatment aims to restore a painless, minimally shortened digit with durable and sensate skin with preserved function, a satisfactory aesthetic outcome and will take into account patient preferences for tip length and speed of return to work [1], [5], [6].

Microsurgical replantation may play an important part in the treatment of distal fingertip amputations, in some cases salvaging the tip, resulting in superior functional and aesthetic outcomes [7], [8], [9]. Arterial or venous anastomoses, however, are impossible at very distal levels [7], especially in the paediatric population where vessels are smaller, and in some crush and avulsion amputations.

The Ishikawa classification adapted to distal fingertip amputations categorises amputations in terms of zones of the fingertip based on the nail. It comprises four zones distal to the DIPJ and takes into account the angle of the amputation [10]. Microsurgery requires the appropriate equipment (microscope and set), post-operative monitoring and set-up and motivated patients. It is associated with high operation costs, prolonged operative time and inpatient stay.

Composite grafting, where the amputated tip is directly sutured onto the proximal stump as a composite graft, is an alternative option for a non-replantable amputated tip. The tip is initially nourished by diffusion, and later through neovascularisation. Composite grafting is a simple time- and cost-effective technique. It may preserve digital length, in some cases restore sensory and motor function and a near-normal nail complex, using durable and glabrous soft tissue coverage and uses the patient’s own tissue in its normal location which results in cosmetically pleasing results [11]. It also avoids the need for microsurgery and the donor site morbidity inherent with flaps.

Composite grafting has been widely performed for distal fingertip amputations but variable success rates are reported through-out the literature with the key complications being infection and necrosis [11], [12], [13], [14], [15], [16], [17], [18], [19], [20], [21], [22], [23], [24], [25], [26], [27]. This has led to scepticism especially surrounding its use in adults [13], [14], [28]. There is additional controversy as to which factors are especially influential on composite grafting success, such as the amputation-reattachment delay, amputation mechanism and level. There have been multiple previous case series documenting composite graft outcomes, but no formal synthesis of results. Therefore, a systematic review will be conducted to understand the indications, functional and aesthetic outcomes, complications, secondary surgery and factors associated with the success of composite grafting for fingertip amputation. We hope such a review will help guide evidence-based practice.

## 2 Methods

This systematic review will be conducted in line with the Cochrane Handbook for Systematic Reviews and Interventions [29] and is compliant with PRISMA guidelines [30]. A systematic review protocol will be published (http://www.ijsprotocols.com/) and the systematic review will be registered a priori: http://www.researchregistry.com/. Both protocol registration and publication will be openly accessible.

### 2.1 Criteria

#### 2.1.1 Studies included

Original research studies of levels 1–5 of the Oxford Centre for Evidence-Based Medicine [31] will be considered for inclusion if reporting data concerning the relevant outcomes, as well as unpublished data, if methods and data are accessible. No duplicate articles nor articles not reporting primary data will be included.

#### 2.1.2 Participants

The patient population will include children and adults receiving non-microsurgical replantation following distal finger tip amputations, with the aim of reviewing outcomes in these cases in order to elucidate the role of non-microsurgical replantation in the management of distal finger amputations.

#### 2.1.3 Intervention

The interventions included will be composite grafting of the distal tip via non-microsurgical methods following fingertip amputation. Any studies in which microsurgical reconstruction is used will not be included. Articles will be included if they report on the survival outcomes of distal fingertip amputations treated with primary composite grafting of the amputated tip. All articles using subcutaneous pocket techniques, ‘pulp flaps’ or microsurgical replantation will be excluded, as will articles reporting on data of less than five cases, following previous research [9].

#### 2.1.4 Comparators

Not applicable.

## 3 Outcomes

The primary outcome measured will be graft survival. Secondary outcomes will include:

• Follow up period (mean and total)
• Reported adverse outcomes, including revision surgery
• Findings of any additional actors associated with graft survival (e.g, age, smoking, diabetes)
• Sensory outcomes
• Functional outcomes
• Aesthetic outcomes

## 4 Search methods and search terms

An electronic database search will be conducted on OVID Medline, PubMed, EMBASE, SCOPUS, The Cochrane Library and clinical trial registries using the terms “fingertip” “fingertips” “digital tip” “digital tips” “digit” “digits” “finger” “fingers” “thumb” “thumbs” “amputation” “amputations” “injury” “injuries” “replantation” “replantations” “reattachment” “reattachments” “reimplantation” “reimplantations” “composite graft” “composite grafts” as keywords combined with the Boolean logical operators “OR” and “AND”. The search is limited to English studies and studies conducted in humans. Duplicated studies will be removed.

## 5 Identification and selection of studies

Two independent reviewers (MRB and MLL) will screen the title and abstract of each of the published articles for inclusion according to the criteria listed in Table 1. Full-length manuscripts will be reviewed for articles which meet the inclusion criteria, if no abstract is published or if the abstract does not have sufficient information to determine eligibility.

**Table 1:** Study inclusion and exclusion criteria.

*Inclusion criteria*
• Primary data
• Outcomes of ‘composite grafts’ or ‘non-microsurgical replantation’ of the amputated part
• Graft survival
• Report on ≥5 cases
• Articles written in English

*Exclusion criteria*
• Composite graft pocketing
• Microsurgical vascular anastomosis
• Use of additional skin flaps or pulp flaps
• Incomplete data
• Cases of composite graft as a secondary revision

## 6 Data extraction, collection and management

Two independent researchers (MRB and MLL) will perform data extraction for each article independently, and studies included will be cross-checked. Data will be entered directly into a pre-formatted database with standardised extraction fields (Microsoft Excel Version 15.23, 2016, Microsoft). If two articles reported on the same data only the higher quality one will be kept.

Data extracted from each article will include: details on study authors; title; journal of publication; date of publication; geographical origin of the research. The demographic details will include patient number, mean age, number of digits, number of males and females, amputation mechanism and level, amputation level classification method, operative details and comorbidities. The outcomes extracted will include: graft survival (%), graft survival definition, adverse outcomes including revision surgery, functional, sensory and cosmetic outcomes and how they were measured. These are shown in Table 2. The level of evidence will be assessed and classified according to the Levels of Evidence table published by the Centre for Evidence Based Medicine [31].

**Table 2:** Details extracted from each study.

*Demographic details*
• Title
• Journal
• Authors
• Country of origin of research
• Level of evidence
• Email of the author of correspondence

*Population details*
• Number of patients
• Number of males/females
• Number of digits amputated
• Amputation mechanism: *avulsion-crush, cut, other*
• Classification scheme used for amputation level: *Ishikawa, modified-Ishikawa, Allen, Hirase, other*

*Perioperative details*
• Modifications to the classic composite graft technique (defatting, proximal stump trimming, bone excision)
• Preservation/removal of the nail bed
• Method of anaesthesia
• Postoperative splinting
• Postoperative antibiotic use
• Postoperative cooling

O*utcomes measured*
• Follow up period (mean and total)
• Graft survival:
o Definition
o When measured, how measured
o % of patients with graft *complete survival, partial survival, failure*
• Reported adverse outcomes, including revision surgery
• Findings of any additional factors associated with graft survival
o Age
o Smoking
o Time lag to surgery
o Diabetes
• Sensory outcomes
o Method used to measure: *questionnaire, 2-point discrimination*
o Reported sensory findings
• Functional outcomes
o Method used to measure: *questionnaire, clinician reported*
o Findings
• Aesthetic outcomes
o Measured: *questionnaire, clinician reported*
o Aesthetic outcomes reported
Any additional outcomes reported

## 7 Data analysis

Summary statistics will be reported as ranges. A weighted mean for each outcome based on sample size of each study will be calculated using Microsoft Excel Software (Version 15.23, 2016, Microsoft).

### 7.1 Subgroup analysis

Analysis of results according to the classification of amputation (proximal or distal), patient age and type of amputation will be performed.

### 7.2 Heterogeneity

Inter-study heterogeneity will be explored for each variable using the Chi square statistic. I2 values will be calculated to quantify the degree of heterogeneity across trials that could not be attributed to chance alone. Significant heterogeneity will be considered present when I2 > 50%. Two strategies will be used to assess data validity and heterogeneity; 1) funnel plots to evaluate publication bias and, 2) a subgroup analysis of higher quality studies (studies with quality scores >10).

### 7.3 Quality scoring

The Grading of Recommendation Assessment, Development and Evaluation (GRADE) system will be used to assess the methodological quality of included studies. The GRADE system offers four levels of evidence: high; moderate; low; very low. RCTs are considered highest level of evidence. Case series and case reports are ‘very low’. Quality may be downgraded along five domains: 1) Study design or implementation limitations; 2) Inconsistency in results; 3) Indirectness of evidence; 4) Imprecision of estimates; and 5) Publication bias. Quality may be upgraded because of three domains: 1) A very large magnitude of effect; 2) A dose-response gradient; 3) All plausible biases would reduce an apparent treatment effect. For RCTs it will be documents: 1) whether or not clinically relevant outcomes are reported; 2) whether results are comparable with protocols and subsequent publications where available. Key missing information across all study types will be documented and assessed.

### 7.4 Assessment of bias

Risk of bias will be assessed using the Cochrane risk of bias tool [32]. All included articles will be subjectively reviewed and assigned a value of “yes,” “no,” or “unclear” to the following questions: (i) Was the allocation sequence adequately generated? (ii) Was allocation adequately concealed? (iii) Was there blinding of participants, personnel, and outcome assessors? (iv) Were incomplete outcome data sufficiently assessed? and (v) Are reports in the study free of the suggestion of selective outcome reporting? Risk of bias plots will be generated.

## 8 Dissemination

The manuscript of this review will be published in a peer-reviewed journal and results will be presented at national and international conferences to inform the practice of other clinicians in the management of distal fingertip amputations.

## 9 Authors’ contributions

MRB and AG conceived this paper. MRB and MLL drafted the article, and all authors critically revised it for important intellectual content, and approved the final version for publication.

## Funding

The research received no specific grant from any funding agency in the public, commercial or non-for-profit sectors.

## Declaration of Competing Interest

The authors have no competing interests to declare.

## References

[1] Fassler P.R. (1996). Fingertip injuries: evaluation and treatment. J. Am. Acad. Orthopaedic Surg..

[2] Fetter-zarzeka A., Joseph M.M. (2002). Hand and fingertip injuries in children. Pediatr. Emerg. Care.

[3] Gellman H. (2009). Fingertip-nail bed injuries in children: current concepts and controversies of treatment. J. Craniofacial Surg..

[4] Doraiswamy N., Baig H. (2000). Isolated finger injuries in children – incidence and aetiology. Injury.

[5] Lemmon J.A., Janis J.E., Rohrich R.J. (2008). Soft-tissue injuries of the fingertip: methods of evaluation and treatment. An algorithmic approach. Plast. Reconstr. Surg..

[6] Martin C., del Pino J.G. (1998). Controversies in the treatment of fingertip amputations: conservative versus surgical reconstruction. Clin. Orthop. Relat. Res..

[7] Hattori Y. (2007). Fingertip replantation. J. Hand Surg..

[8] Sebastin S.J., Chung K.C. (2011). A systematic review of the outcomes of replantation of distal digital amputation. Plast. Reconstr. Surg..

[9] Wang K. (2013). A systematic review of outcomes of revision amputation treatment for fingertip amputations. Hand.

[10] Evans D.M., Bernadis C. (2000). A new classification for fingertip injuries. J. Hand Surg..

[11] Eo S. (2009). Successful composite graft for fingertip amputations using ice-cooling and lipo-prostaglandin E1. J. Plastic, Reconstr. Aesthetic Surg..

[12] Douglas B. (1959). Successful replacement of completely avulsed portions of fingers as composite grafts. Plast. Reconstr. Surg..

[13] Moiemen N., Elliot D. (1997). Composite graft replacement of digital tips 2. A study in children. J. Hand Surg. (British and European Volume).

[14] Heistein J.B., Cook P.A. (2003). Factors affecting composite graft survival in digital tip amputations. Ann. Plast. Surg..

[15] Hirase Y. (1993). Postoperative cooling enhances composite graft survival in nasal-alar and fingertip reconstruction. Br. J. Plast. Surg..

[16] Butler D. (2015). The outcomes of digital tip amputation replacement as a composite graft in a paediatric population. J. Hand Surg. (European Volume).

[17] Kiuchi T. (2015). Composite grafting for distal digital amputation with respect to injury type and amputation level. J. Plastic Surg. Hand Surg..

[18] Rose E.H. (1989). The “cap” technique: nonmicrosurgical reattachment of fingertip amputations. J. Hand Surg..

[19] Chen S.-Y. (2011). Composite grafting for traumatic fingertip amputation in adults: technique reinforcement and experience in 31 digits. J. Trauma Acute Care Surg..

[20] Dagregorio G., Saint-Cast Y. (2006). Composite graft replacement of digital tips in adults. Orthopedics.

[21] Kankaya Y. (2006). An alternative technique for microsurgically unreplantable fingertip amputations. Ann. Plast. Surg..

[22] Kusuhara H. (2011). Randomized controlled trial of the application of topical b-FGF-impregnated gelatin microspheres to improve tissue survival in subzone II fingertip amputations. J. Hand Surg. (European Volume).

[23] Murphy A.D. (2016). Paediatric fingertip composite grafts: do they all go black?. J. Plastic, Reconstr. Aesthetic Surg..

[24] Imaizumi A. (2013). Validity of exploration for suitable vessels for replantation in the distal fingertip amputation in early childhood: replantation or composite graft. J. Plastic Surg. Hand Surg..

[25] Son D., Han K., Chang D.W. (2005). Extending the limits of fingertip composite grafting with moist-exposed dressing. Int. Wound J..

[26] Urso-Baiarda F.G., Wallace C.G., Baker R. (2009). Post-traumatic composite graft fingertip replantation in both adults and children. Eur. J. Plast. Surg..

[27] Eberlin K.R. (2008). Quality assurance guidelines for surgical outreach programs: a 20-year experience. Cleft Palate-Craniofacial J..

[28] Adani R., Marcoccio I., Tarallo L. (2003). Treatment of fingertips amputation using the Hirase technique. Hand Surg..

[29] Higgins J.P., Green S. (2008). Cochrane Handbook for Systematic Reviews of Interventions.

[30] Moher D. (2009). Preferred reporting items for systematic reviews and meta-analyses: the PRISMA statement. Ann. Intern. Med..

[31] Oxford Centre for Evidence-based Medicine - Levels of ... (n.d.). Retrieved from https://www.cebm.net/2009/06/oxford-centre-evidence-based-medicine-levels-evidence-march-2009/.

[32] Chapter 8: Assessing risk of bias in included studies. (n.d.). Retrieved from http://handbook-5-1.cochrane.org/chapter_8/8_assessing_risk_of_bias_in_included_studies.htm.

